# Auditory Resource Redistribution in Audiovisual Integration: Evidence from Attribute Amnesia

**DOI:** 10.3390/bs15111557

**Published:** 2025-11-14

**Authors:** Zikang Meng, Ziyi Liu, Wu Jiang, Biye Cai, Zonghao Zhang, Haoping Yang

**Affiliations:** 1 School of Physical Education and Sports Science, Soochow University, Suzhou 215021, China; 20244206035@stu.suda.edu.cn (Z.M.); 20244206019@stu.suda.edu.cn (Z.L.); 20254206036@stu.suda.edu.cn (W.J.); 20234006005@stu.suda.edu.cn (B.C.); 2 School of Psychology, Beijing Sport University, Beijing 100091, China

**Keywords:** attribute amnesia, audiovisual integration, attentional blink, working memory reselection, semantic processing

## Abstract

Auditory stimuli are known to enhance visual target recognition in rapid serial visual presentation (RSVP) tasks, yet the robustness and potential trade-offs of this audiovisual integration (AVI) effect remain debated. Attribute amnesia (AA) refers to the phenomenon in which individuals successfully identify a stimulus for a task, but fail to recall its basic attributes when unexpectedly tested. The present study investigates whether improvements in visual recognition through AVI occur at the expense of auditory information loss, as predicted by the AA framework. Across two RSVP experiments, participants were presented with letter targets embedded among digit distractors. In Experiment 1, an auditory pitch (bass, alto, treble) accompanied the second target (T2); in Experiment 2, an auditory syllable either matched or mismatched the semantic identity of T2. A surprise-test paradigm was used to assess participants’ ability to recall auditory stimuli. The results show that both pitch and semantic attributes were subject to AA, with semantic stimuli recalled more accurately than pitch. Moreover, semantic congruency enhanced T2 identification, highlighting the automatic processing advantage of semantic cues. Post-surprise trials revealed the improved recall of auditory attributes, consistent with the working memory reselection model. Together, these findings suggest that AVI enhances visual recognition by reallocating cognitive resources, but at the cost of a partial loss of irrelevant auditory information.

## 1. Introduction

Rapid serial visual presentation (RSVP) tasks often reveal attentional blink (AB), a temporary reduction in detecting a second target (T2) when it follows the first target (T1) within a short interval (Raymond et al., 1992). This limitation is typically attributed to a processing bottleneck caused by the allocation of limited attentional resources (Jolickur, 1999; Shapiro et al., 1994; Pant, 2018; Shimojo & Shams, 2001). Thus, AB provides a well-established framework for examining how visual attention is distributed across time.

Although AB is a visual phenomenon, auditory signals can facilitate visual target recognition. This effect is broadly referred to as audiovisual integration (AVI) (Asakawa et al., 2011; Molholm et al., 2002; Reiner, 2009; Talsma et al., 2010; Zhou et al., 2020). In RSVP paradigms, auditory stimulation consistently enhances T2 identification (Aijun et al., 2022; H. Yang et al., 2023; Zhao et al., 2021b; Zhao et al., 2023; Zhao et al., 2022). Two accounts have been proposed: the shared-resource hypothesis suggests that multimodal inputs increase efficiency (Stefanics et al., 2010), whereas the resource-transfer hypothesis states that auditory resources can be redistributed to support visual processing. Both perspectives assume that cognitive resources are limited and may shift across modalities.

Attention plays a key role in selecting features for working memory. Irrelevant attributes are often degraded to preserve resources for task-relevant information (Awh et al., 2006). The working memory reselection model further proposes that multiple latent representations exist and that unattended attributes may remain imprecise until recall demands trigger their re-selection (Hedayati et al., 2022). These models suggest that increased visual performance under AVI may lead to reduced retention of auditory features. However, direct empirical evidence of such a trade-off is limited.

Findings on the automaticity of auditory and semantic processing provide additional complexity. Some studies report that AVI emerges automatically and is unaffected by semantic load (Zhao et al., 2021a, 2021b, 2022; Aijun et al., 2022; H. Yang et al., 2024; Zhou et al., 2020). Other work indicates that semantic information can interfere with or inhibit visual recognition, particularly under higher attentional demands (Sulpizio et al., 2024; H. Yang et al., 2023). Attending to auditory stimuli further modulates AVI (Lunn et al., 2019). Cross-modal conflict can slow responses (Misselhorn et al., 2019), and inconsistent auditory semantics may impair visual target detection (Misselhorn et al., 2019). Evidence regarding pitch is also mixed: some studies report no pitch-related modulation of AVI (Aijun et al., 2022), whereas others identify asymmetric audiovisual effects, with auditory information exerting a stronger influence on visual processing compared to the reverse (H. Yang et al., 2024).

Despite substantial progress, an important question remains unaddressed: Does AVI enhance visual target recognition by sacrificing auditory information, as predicted by the attribute amnesia (AA) framework? AA refers to the failure to recall basic attributes of a task-relevant stimulus when unexpectedly tested, even when the stimulus was successfully processed for the primary task. This paradigm provides a sensitive method for identifying which auditory attributes are down-weighted during cross-modal processing.

To address this gap, the present study combined an RSVP paradigm with a surprise-test AA procedure. We examined whether auditory pitch (Experiment 1) and auditory semantic information (Experiment 2) are susceptible to AA when paired with T2. We also assessed whether semantic information, which often undergoes more automatic processing, would show greater resistance to AA than pitch.

We formulated the following hypotheses:

**H1.**  *Auditory pitch information would show AA, with lower recall accuracy at shorter temporal lags.*

**H2.**  *Semantic auditory information would also show AA, but would be recalled more accurately than pitch.*

**H3.**  *Semantic congruency between auditory input and T2 would enhance T2 recognition.*

**H4.**  *Congruent semantic cues would facilitate recall of auditory information.*

By integrating AVI, attentional resource theories, and AA, the present work aims to clarify how the cognitive system prioritizes and discards auditory attributes during audiovisual processing.

## 2. Methods and Materials

The present study included two RSVP experiments designed to investigate whether auditory pitch (Experiment 1) and auditory semantic information (Experiment 2) are subject to attribute amnesia (AA). Both experiments employed a surprise-test paradigm in which participants were unexpectedly asked to recall auditory attributes after performing the primary visual task. The experiments shared the same basic RSVP structure, with visual distractors and targets presented in rapid succession while auditory stimuli were paired with the second target (T2) (Wang et al., 2025). To ensure methodological rigor, sample size estimation was conducted prior to recruitment, and all procedures followed ethical standards approved by the institutional ethics committee.

### 2.1. Participants

Experiment 1 adopted a 2 (lag: lag 3 vs. lag 8) × 3 (pitch: bass, alto, treble) design, while Experiment 2 used a 2 (lag: lag 3 vs. lag 8) × 2 (semantic consistency: consistent vs. inconsistent) design. Sample size estimation was conducted with G*Power 3.1.9.2 (Faul et al., 2007), based on a medium effect size (f = 0.25) for repeated-measures ANOVA designs (2 × 2 or 2 × 3), with α = 0.05 and power = 0.90. The analysis indicated that at least 30 participants were required for each experiment. A total of 86 healthy adults were recruited (Mage = 25.4 years, SD = 2.8; 42 female). All participants were right-handed, reported normal or corrected-to-normal vision and normal hearing, and had no history of psychiatric or neurological disorders. Recruitment was conducted through online platforms and community posters.

Before participation, all individuals provided written informed consent and received monetary compensation of approximately USD $15 per hour. Data from six participants were excluded: five were unable to reliably discriminate auditory stimuli, and one produced insufficient visual responses. The final dataset comprised 39 participants in Experiment 1 (17 male, 22 female; Mage = 20.6 years, SD = 1.2; age range: 18–22) and 41 participants in Experiment 2 (19 male, 22 female; Mage = 20.8 years, SD = 1.3; age range: 18–22). All procedures were conducted in accordance with the Declaration of Helsinki and were approved by the Ethics Committee of Beijing Sport University (Approval No. 2024349H).

### 2.2. Experimental Procedures and Design

All experiments were conducted in a quiet laboratory. Visual stimuli were presented on a 19-inch Dell E2316HF monitor with a resolution of 1280 × 1024 pixels and a refresh rate of 60 Hz. Participants were seated approximately 80 cm from the screen. Stimuli were controlled and presented using MATLAB 2019a with Psychtoolbox 3.1.2 (Kleiner et al., 2007).

Each RSVP stream contained 17 items, displayed for 67 ms with a 33 ms blank interval, yielding a stimulus onset asynchrony (SOA) of 100 ms. A fixation cross appeared for 1000 ms at the start of each trial. T1 was randomly positioned between the 5th and 9th items, and T2 appeared either three items (lag 3) or eight items (lag 8) after T1. Distractors were Arabic numerals (0–9) arranged in a pseudorandom odd–even alternating sequence to prevent immediate repetition. Target stimuli were uppercase Latin letters, with easily confusable letters (e.g., O, I, Q) excluded. Participants were informed that letters served as targets, but were not told which specific letters would appear as T1 or T2.

Each experimental session had three phases. In the pre-surprise phase, participants completed 70 standard trials, reporting T1 and T2 after each stream. The surprise trial (trial 71) followed the same format, but additionally required recall of the auditory stimulus (T3). In Experiment 1, participants judged whether the tone was low, middle, or high; in Experiment 2, they identified the vowel and indicated whether it was congruent with T2. The post-surprise phase then included 10 trials identical to the surprise trial, allowing for the assessment of adaptation once participants realized auditory information could be tested. All surprise and post-surprise trials were administered under the lag 3 condition.

**Figure 1:**
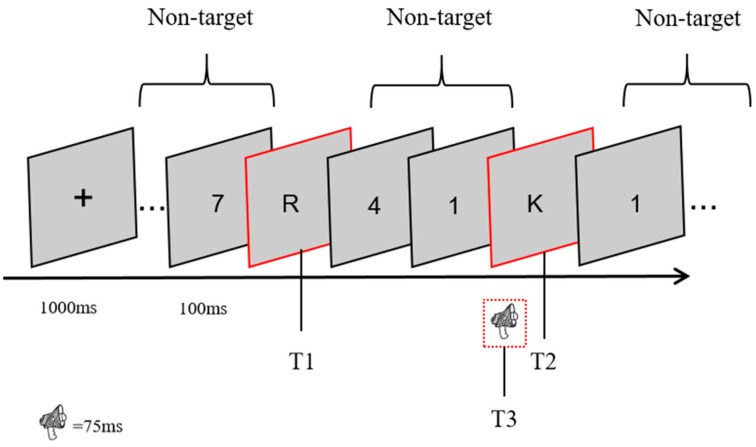
An RSVP experiment. Subjects were required to report T1 and T2 in the task and to report T3 in surprise test and post-surprise test. In Experiment 1, the T3 stimulus consisted of pure B pitches of different keys (bass B4 (494 Hz), alto B5 (988 Hz), and treble B6 (1976 Hz)), while in Experiment 2, the T3 stimulus consisted of monosyllabic vowels that either agreed or disagreed with T2. All of the surprise tests occurred under lag3 conditions.

Before the formal task, participants completed practice trials to become familiar with the procedure. The entire session, including practice, lasted approximately 30–40 min. The overall design of the RSVP task and the corresponding surprise test procedure are illustrated in Figure 1.

#### 2.2.1. Auditory Stimuli

For Experiment 1, auditory stimuli consisted of pure B tones presented at three different pitch levels: bass (B4, 494 Hz), alto (B5, 988 Hz), and treble (B6, 1976 Hz) (H. Yang et al., 2023). Each tone lasted 75 ms, followed by a 25 ms silent gap. The presentation level was fixed at approximately 75 dB SPL. In Experiment 2, auditory stimuli consisted of monosyllabic vowels recorded by a native English speaker. Each vowel was standardized for intensity and duration and paired with either a letter matching the vowel (e.g., /eɪ/paired with the letter A) or a letter mismatching the vowel (H. Yang et al., 2024).

#### 2.2.2. Auditory-Visual Congruency

In Experiment 1, pitch congruency was defined by pairing the auditory stimuli with the visual targets (letters) in a congruent or incongruent manner. In the congruent condition, the pitch of the auditory stimuli was selected based on the semantic association of the letter, and the matching auditory pitch was pre-rated by participants in a pilot study for semantic relevance. In the incongruent condition, the auditory pitch was paired with a visually inconsistent target, introducing cross-modal interference.

For Experiment 2, semantic congruency was defined by the pairing of monosyllabic vowels with visually congruent or incongruent letters. Semantic congruency was validated through pilot ratings of the visual and auditory pairings to ensure meaningful congruency and incongruency. Participants rated the semantic relevance of each pairing, confirming that the stimuli used were appropriately congruent or incongruent. To ensure proper synchronization of auditory and visual stimuli, the auditory stimulus was presented simultaneously with the visual target (T2). The auditory-visual timing was calibrated during a pilot session to ensure that participants perceived the stimuli as synchronized. In addition, loudness calibration was conducted for both pitch and semantic congruency conditions to ensure equivalent perceptual salience across auditory stimuli. Participants were asked to rate the auditory-visual alignment and perceived intensity before the main experiment, ensuring that no perceptual biases influenced the results.

The AA surprise-test procedure was used to assess the effects of attribute amnesia (AA) on participants’ ability to recall auditory stimuli following the RSVP task. After completing the main task (reporting T1 and T2), participants were unexpectedly asked to recall the auditory stimulus (T3) from the trial. This procedure was designed to prevent participants from anticipating the auditory recall task and to minimize strategic encoding.

### 2.3. Data Analysis

Behavioral accuracy data for both dual-target (T2T1) and auditory-recall (T3) tasks were analyzed across the three experimental phases: pre-surprise, surprise, and post-surprise.

Confirmatory analyses were performed for the pre-surprise phase using a 2 (Lag: 3, 8) × 3 (Auditory Condition: Pitch, Semantic-consistent, Semantic-inconsistent) repeated-measures ANOVA. Normality and sphericity assumptions were checked (Shapiro–Wilk > 0.05; Mauchly’s test > 0.05); Greenhouse–Geisser correction was applied when violated. Exploratory analyses were conducted for the surprise and post-surprise phases: chi-square tests examined between-experiment T3 differences, and non-parametric Wilcoxon signed-rank tests compared T2|T1 and T3 across conditions due to non-normal distributions (*p* < 0.05).

All tests were two-tailed with *α* = 0.05, and Bonferroni corrections were applied for multiple comparisons. Effect sizes were reported as partial $\eta_{p}^{2}$ (for ANOVA) and Cohen’s *d* (for pairwise comparisons), accompanied by 95% confidence intervals. Participants responded via keyboard without time limits, and audiovisual pairings were fully counter-balanced. Analyses were conducted in Python (3.9) using Pingouin (version 0.5.1) and Statsmodels (version 0.13.2) libraries.

## 3. Results

We analyzed the correct rate of T2T1 and T3 across the three phases of the experiment. Descriptive statistics are summarized in Table 1.

**Figure 2:**
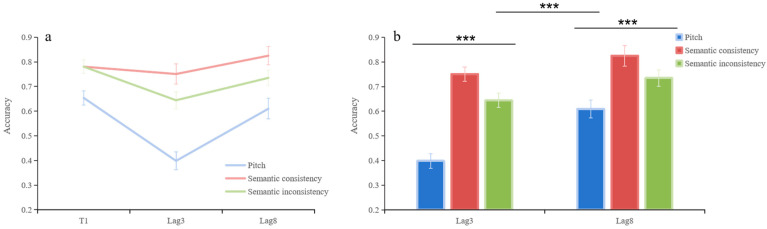
In the pre-surprise test, T2T1 results under different auditory stimuli and lag conditions. (**a**) Changes in different auditory stimuli conditions. (**b**) Comparison of different lag and auditory stimuli conditions. The error line is the standard error. *** *p* < 0.001.

In pre-surprise test, T2T1 is influenced by lag and auditory stimuli (Figure 2a). The results showed a significant lag main effect, *F*(1, 38) = 34.52, *p* < 0.001, $\eta_{p}^{2}$ = 0.48. A significant main effect of auditory stimuli was observed, *F*(2, 76) = 59.85, *p* < 0.001, $\eta_{p}^{2}$ = 0.61. The relationship between the lag and auditory stimuli exhibited statistical significance, *F*(2, 76) = 8.79, *p* = 0.001, $\eta_{p}^{2}$ = 0.19.

Simple effect analysis was performed with lag and auditory stimuli as the main factors (Figure 2b). In lag3, a significant difference observed between pitch and conditions in which auditory stimuli are inconsistent with T2 semantics (*t* = −7.69, *p* < 0.001, Cohen’s *d* = −1.01, 95%CI = [−0.311, −0.180]); a significant difference observed between pitch and conditions in which auditory stimuli are consistent with T2 semantics (*t* = −13.04, *p* < 0.001, Cohen’s *d* = −1.57, 95%CI = [−0.408, −0.297]); a significant difference observed between conditions in which auditory stimuli are consistent with T2 semantics and conditions in which auditory stimuli are inconsistent with T2 semantics (*t* = 4.46, *p* < 0.001, Cohen’s *d* = 0.45, 95%CI = [0.058, 0.155]); In lag8, a significant difference observed between pitch and conditions in which auditory stimuli are inconsistent with T2 semantics (*t* = −3.13, *p* < 0.001, Cohen’s *d* = −0.5, 95%CI = [−0.205, −0.045]); a significant difference observed between pitch and conditions in which auditory stimuli are consistent with T2 semantics (*t* = −5.38, *p* < 0.001, Cohen’s *d* = −0.94, 95%CI = [−0.296, −0.134]); a significant difference observed between conditions in which auditory stimuli are consistent with T2 semantics and conditions in which auditory stimuli are inconsistent with T2 semantics (*t* = 3.91, *p* < 0.001, Cohen’s *d* = 0.42, 95%CI = [0.043, 0.137]).

Bonferroni-corrected simple effect analyses were conducted with lag and auditory stimuli as the main factors (Figure 2b). At Lag 3, accuracy in the semantic-consistent condition was significantly higher than in the pitch condition (*t* = −13.04, *p* < 0.001, Cohen’s *d* = −1.57, 95% CI = [−0.408, −0.297]) and the semantic-inconsistent condition (*t* = 4.46, *p* < 0.001, Cohen’s *d* = 0.45, 95% CI = [0.058, 0.155]). The semantic-inconsistent condition also showed higher accuracy than the pitch condition (*t* = −7.69, *p* < 0.001, Cohen’s *d* = −1.01, 95% CI = [−0.311, −0.180]). At Lag 8, a similar pattern emerged. The semantic-consistent condition outperformed the pitch condition (*t* = −5.38, *p* < 0.001, Cohen’s *d* = −0.94, 95% CI = [−0.296, −0.134]) and the semantic-inconsistent condition (*t* = 3.91, *p* <0.001, Cohen’s *d* = 0.42, 95% CI = [0.043, 0.137]). Accuracy in the semantic-inconsistent condition was also significantly higher than in the pitch condition (*t* = −3.13, *p* < 0.001, Cohen’s *d* = −0.50, 95% CI = [−0.205, −0.045]).

In pitch condition, a significant difference observed between lag3 and lag8 (*t* = −7.28, *p* < 0.001, Cohen’s *d* = −0.87, 95% CI = [−0.271, −0.152]); accuracy at Lag 8 remained significantly higher than at Lag 3 (*t* = –7.28, *p* < 0.001, Cohen’s *d* = 0.87, 95% CI [–0.271, –0.152]). In conditions in which auditory stimuli are consistent with T2 semantics, a significant difference observed between lag3 and lag8 (*t* = −2.55, *p* = 0.014, Cohen’s *d* = 0.37, 95% CI = [−0.133, −0.016]); In conditions in which auditory stimuli are inconsistent with T2 semantics, a significant difference observed between lag3 and lag8 (*t* = −2.94, *p* = 0.006, Cohen’s *d* = 0.37, 95% CI = [−0.154, −0.028]). The same pattern was observed for the semantic-consistent (*t* = –2.55, *p* = 0.014, Cohen’s *d* = 0.37, 95% CI [–0.133, –0.016]) and semantic-inconsistent conditions (*t* = –2.94, *p* = 0.006, Cohen’s *d* = 0.37, 95% CI [–0.154, –0.028]), indicating that prolonged temporal separation improved detection across all auditory types.

**Figure 3:**
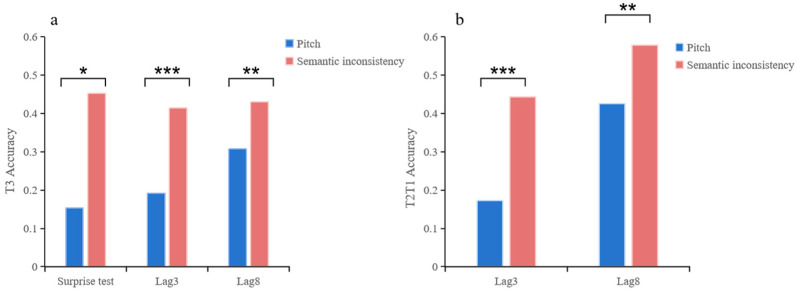
T3 and T2T1 results. (**a**) T3 results under different auditory stimuli and lag conditions. (**b**) T2T1 results under different auditory stimuli and lag conditions. * *p* < 0.05, ** *p* < 0.01, *** *p* < 0.001.

In the surprise test, there is a significant difference in T3 between Experiment 1 (15.38%) and Experiment 2 (46.15%) (Figure 3a), *χ^2^* (1, *N* = 39) = 4, *p* = 0.012, *φ* = 0.32.

In the post-surprise test, there is a significant difference in T2T1 between Experiment 1 and Experiment 2 (Figure 3b). In lag3, Experiment 1 (P_25_ = 0.1, P_50_ = 0.2, P_75_ = 0.3) is significantly lower than Experiment 2 (P_25_ = 0.3, P_50_ = 0.4, P_75_ = 0.5), *p* < 0.001, z = 5.02. In lag8, Experiment 1 (P_25_ = 0.3, P_50_ = 0.5, P_75_ = 0.6) is significantly lower than Experiment 2 (P_25_ = 0.5, P_50_ = 0.6, P_75_ = 0.6), *p* = 0.001, z = 3.28.

In the post-surprise test, there is a significant difference in T3 between Experiment 1 and Experiment 2 (Figure 3a). In lag3, Experiment 1 (P_25_ = 0.1, P_50_ = 0.2, P_75_ = 0.3) is significantly lower than Experiment 2 (P_25_ = 0.3, P_50_ = 0.4, P_75_ = 0.4), *p* < 0.001, z = 4.54. In lag8, Experiment 1 (P_25_ = 0.2, P_50_ = 0.3, P_75_ = 0.4) is significantly lower than Experiment 2 (P_25_ = 0.3, P_50_ = 0.4, P_75_ = 0.5), *p* = 0.002, z = 3.11.

In the post-surprise phase, both dual-target (T2T1) and auditory-recall (T3) performance remained significantly higher in Experiment 2 compared with Experiment 1 (Figure 3). Bonferroni-corrected Wilcoxon signed-rank tests revealed that participants in Experiment 2 maintained superior accuracy across both lag conditions.

For T2T1, Experiment 2 outperformed Experiment 1 at Lag 3 (*z* = 5.02, *p* < 0.001; P25 = 0.10, P50 = 0.20, P75 = 0.30 vs. P25 = 0.30, P50 = 0.40, P75 = 0.50) and Lag 8 (z = 3.28, *p* = 0.001; P25 = 0.30, P50 = 0.50, P75 = 0.60 vs. P25 = 0.50, P50 = 0.60, P75 = 0.60).

For T3, a similar trend was observed. Experiment 2 participants demonstrated higher recall accuracy at Lag 3 (*z* = 4.54, *p* < 0.001; P25 = 0.10, P50 = 0.20, P75 = 0.30 vs. P25 = 0.30, P50 = 0.40, P75 = 0.40) and Lag 8 (*z* = 3.11, *p* = 0.002; P25 = 0.20, P50 = 0.30, P75 = 0.40 vs. P25 = 0.30, P50 = 0.40, P75 = 0.50).

## 4. General Discussion

The current study aimed to investigate whether audiovisual integration (AVI) enhances visual target recognition at the cost of auditory information loss, as predicted by the attribute amnesia (AA) framework. Specifically, we explored how auditory pitch and semantic congruency interact with visual processing in the context of the attentional blink (AB). The results confirmed that audiovisual integration leads to enhanced visual accuracy, but this improvement is accompanied by partial loss of auditory information, particularly for non-semantic auditory cues.

The key empirical finding of our study is that while auditory cues—both pitch and semantic—improve visual recognition, they also contribute to a decrease in the accuracy of auditory recall, especially under shorter temporal lags (Lag 3). These results provide direct support for the resource redistribution hypothesis, which posits that cognitive resources are limited and can be reallocated between sensory modalities depending on task relevance (Stefanics et al., 2010). Our data suggest that auditory stimuli, when paired with visual targets, enhance visual processing by reallocating attentional resources, yet this comes at the expense of auditory information retention. This finding is in line with prior research indicating that when attentional resources are focused on visual stimuli, auditory information may be neglected, particularly when it is not directly relevant to the task at hand (Awh et al., 2006; Zhao et al., 2021a).

In Experiment 1, we observed that participants’ recall of auditory pitch information was notably reduced, especially when the lag between the first and second targets (T1 and T2) was short (Lag 3). This result is a typical example of the attentional blink effect, where the second target (T2) suffers from reduced detection accuracy when it follows the first target (T1) closely in time (Raymond et al., 1992). The attentional blink effect can be explained through the concept of a cognitive bottleneck. The bottleneck theory suggests that when cognitive resources are limited, multiple stimuli compete for the same processing resources, allowing for only one target to be processed at a time. When T1 appears, participants’ attention is fully captured by processing T1, consuming a substantial portion of cognitive resources. This leaves insufficient resources to process and encode T2, causing difficulty in recognizing the second target. At short intervals, this competition for cognitive resources limits the processing of T2, leading to reduced recognition accuracy. Despite the addition of auditory stimuli, such as pitch, which enhanced visual recognition, it did not overcome the attentional bottleneck at Lag 3, indicating that even cross-modal audiovisual integration cannot fully alleviate the limitations in resource allocation. This further highlights the inherent limitations of cognitive resources, as participants struggle to process both visual and auditory information simultaneously under these conditions (Petersen & Vangkilde, 2022).

In contrast, at longer temporal lags (Lag 8), when participants had more time to refocus their attention from T1 to T2, the visual recognition of T2 improved significantly, and auditory recall accuracy also increased. This pattern suggests that the duration of the attentional blink can be mitigated with time, allowing for more resources to be dedicated to both visual and auditory processing. The difference in performance between Lag 3 and Lag 8 illustrates the dynamic allocation of cognitive resources based on temporal separation, supporting the notion that the capacity for processing audiovisual stimuli is influenced by attentional demands and time constraints (Pant, 2018).

Beyond the attentional effects, the study also uncovered an interesting difference in the way auditory pitch and semantic information were processed. While both types of auditory stimuli enhanced visual recognition, semantic information was retained more accurately than pitch information in the surprise test. This finding aligns with previous work suggesting that semantic processing is more automatic and less dependent on attentional resources than non-semantic auditory information (Sasin et al., 2023). In Experiment 2, where the auditory stimuli were semantically congruent or incongruent with the visual target, participants were more likely to recall semantically congruent auditory information accurately. This supports the view that semantic processing operates automatically, even when the information is not directly relevant to the primary task (H. Yang et al., 2024; Zhao et al., 2022). The automaticity of semantic processing likely facilitates its encoding into working memory, making it more accessible during recall tasks. This result also suggests that the cognitive system is predisposed to prioritize semantically meaningful information, especially when it is linked with task-relevant visual stimuli.

Our study provides further evidence that semantic congruency between audiovisual stimuli plays a crucial role in both visual and auditory processing. When the auditory information was congruent with the visual target, it not only enhanced visual target recognition, but also improved the recall of the auditory stimulus. This finding is in line with the predictive coding framework, which posits that the brain continuously updates sensory representations based on prior expectations and integrates multisensory information to enhance perception (Peng et al., 2023). In the context of audiovisual integration, congruent auditory information provides a strong predictive cue for visual target recognition, thereby enhancing both the processing and retention of the associated auditory attributes. This effect was particularly pronounced when participants were required to recall auditory stimuli in the surprise test, as the congruent visual target served as a retrieval cue that facilitated the extraction of semantic information from memory.

On the other hand, pitch information did not benefit from this automatic processing advantage. Instead, the processing of pitch was more effortful and required greater cognitive resources. The recall of pitch information was significantly impaired, particularly under conditions of high cognitive load, such as when participants were presented with multiple competing auditory stimuli or when the pitch was incongruent with the visual target. This result may reflect the increased cognitive load associated with pitch discrimination, which involves more complex auditory processing compared to semantic information (Howe & Lee, 2021). In Experiment 1, where participants were asked to identify the pitch of the auditory stimuli, the task required them to categorize the auditory information into discrete categories (bass, alto, treble), which likely increased the cognitive resources required for processing. As a result, participants had fewer resources available for encoding this information into working memory, leading to its subsequent loss during the surprise recall test.

Additionally, our findings underscore the role of automatic semantic processing in shaping the attentional allocation during audiovisual tasks. Previous research has shown that semantic information, even when not directly relevant to the task, can automatically capture attention and modulate sensory processing (Zhao et al., 2021b; H. Yang et al., 2024). In our study, the semantic congruency between auditory and visual stimuli likely facilitated the extraction and retention of relevant auditory features, whereas irrelevant pitch information did not benefit from this automatic processing advantage. The automaticity of semantic processing could explain why participants were able to report semantically congruent auditory information more accurately, even when they had to shift their attention away from the auditory modality to focus on the visual task. This automatic processing mechanism is consistent with theories of attention and working memory, which propose that task-relevant semantic information can be rapidly encoded and retained without requiring conscious effort (X. Yang et al., 2020).

The results of the current study also contribute to the broader understanding of sensory competition and resource allocation within the framework of predictive coding. Predictive coding suggests that the brain continuously integrates sensory inputs and updates internal models based on prior expectations (Peng et al., 2023). In the case of audiovisual integration, the brain combines auditory and visual information to enhance the detection of task-relevant stimuli, while suppressing irrelevant or less salient inputs. This mechanism likely explains why semantic auditory information, which is congruent with the visual target, is more effectively processed and retained, whereas non-semantic auditory cues like pitch are subject to greater attentional interference and are more likely to be forgotten.

This study has several limitations. First, the surprise-test paradigm inherently relies on unexpected recall, which may introduce variability across participants. However, this feature is central to the attribute amnesia framework and does not undermine the overall pattern of results. Second, the present design focused solely on behavioral measures, leaving the underlying neural mechanisms of auditory–visual resource re-distribution unexplored. Future research could employ neurophysiological approaches such as EEG or fMRI to clarify the temporal and neural dynamics of this process. Third, although the results generally supported our hypotheses, we did not observe the predicted decrease in auditory recall accuracy in the semantic-consistent condition, as suggested by hypothesis H4. Instead, the semantic-consistent condition led to higher recall accuracy, which may indicate that audiovisual congruency does not necessarily involve a reallocation of resources that negatively impacts auditory memory. Future studies should explore this hypothesis further, considering different experimental designs and materials that may reveal more about the role of semantic congruency in auditory memory recall. Finally, the sample consisted of healthy young adults. Previous studies have suggested that attribute amnesia may manifest differently in children and older adults compared to young adults, raising questions about the generalizability of the current findings. Extending this line of research to more diverse populations will be critical for determining the robustness and developmental trajectory of cross-modal attribute amnesia.

## 5. Conclusions

This study shows that both the perception of pitch and semantic information occupy auditory channel resources and affect visual information processing. In the surprise test, the participants forgot the pitch and semantic information of the auditory stimulus. In the simultaneous perception of visual and auditory information, the perception and recognition of pitch information require more cognitive resources. Semantic information has the possibility of automatic processing, which shows a higher reporting rate. The evidence that working memory redirects attention to information selection after the surprise test task suggests that the improvement in cross-audiovisual integration is due to the loss of perception of some features of the auditory information.

## Figures and Tables

**Table 1:** Descriptive statistics of accuracy across conditions.

	Variables	Pre-Surprise Test		Surprise Test		Post-Surprise Test	
**Experiment 1**		**Accuracy, Mean (SD)**		**Accuracy**		**Accuracy**	
T2T1	Lag 3	0.40 (0.22)				0.17	
	Lag 8	0.61 (0.26)				0.43	
T3	Lag 3			0.15		0.19	
	Lag 8					0.30	
**Experiment 2**		**Consistent**	**Inconsistent**	**Consistent**	**Inconsistent**	**Consistent**	**Inconsistent**
T2T1	Lag 3	0.75 (0.21)	0.64 (0.26)				0.44
	Lag 8	0.83 (0.19)	0.74 (0.24)				0.58
T3	Lag 3				0.46		0.41
	Lag 8						0.43

Consistent mean that the semantic information of the T3 stimulus was consistent with that of the T2 stimulus. Inconsistent mean that the semantic information of the T3 stimulus was inconsistent with that of the T2 stimulus.

## Data Availability

The data presented in this study are available on request from the corresponding author due to privacy and ethical restrictions.
